# Supplementary material for: Risk Factors, Clinical Characteristics, and Antibiotic Susceptibility Patterns of Streptococcal Keratitis: An 18-Year Retrospective Study from a Tertiary Hospital in China
Source: Antibiotics (Basel). 2024 Dec 6;13(12):1190. doi: 10.3390/antibiotics13121190 (PMC11672442; doi:10.3390/antibiotics13121190)
Supplement: Supplementary file 1 [file antibiotics-13-01190-s001.zip › antibiotics-3290040-supplementary.pdf]

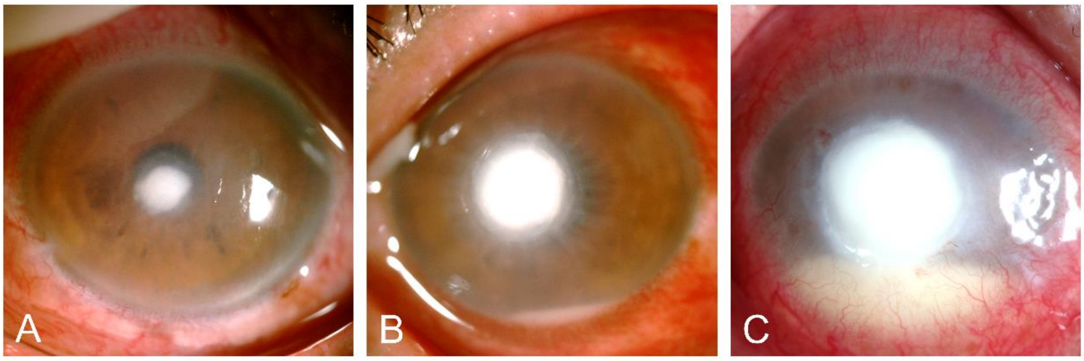

Figure S1: Slit-lamp photographs of different stages of *Streptococcal* keratitis. (A): Stage I; (B): Stage II; (C): Stage III.

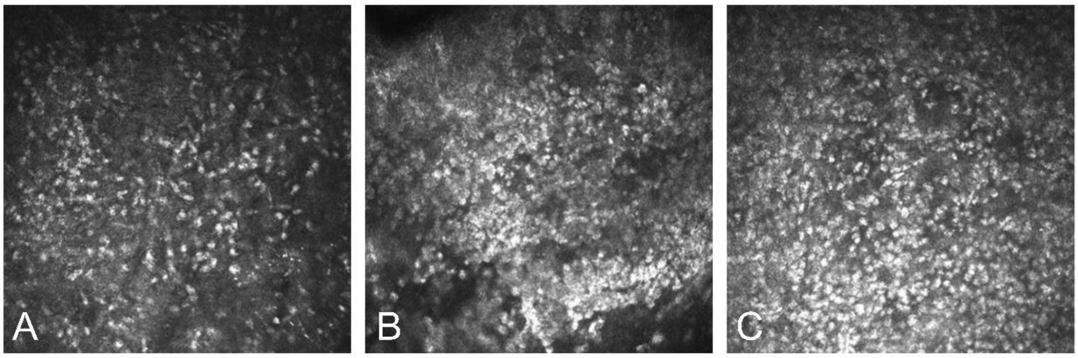

Figure S2: *In vivo* confocal microscopy photographs showing cellular features in the corneal epithelial layer of different stages of *Streptococcal* keratitis. (A): Stage I; (B): Stage II; (C): Stage III.

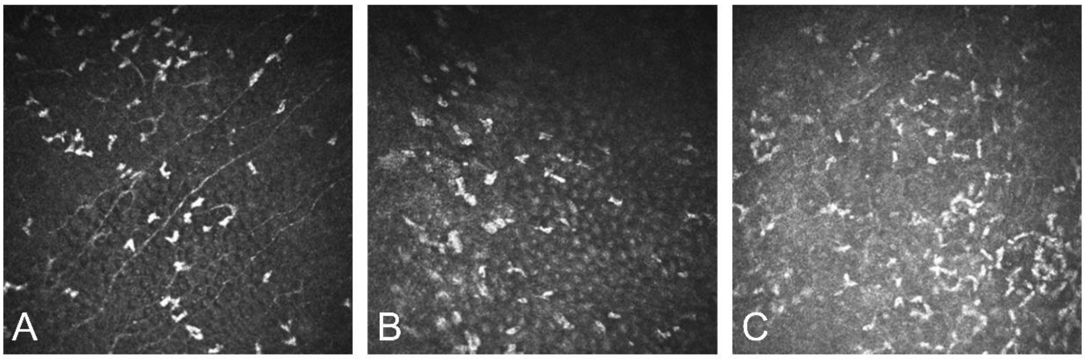

Figure S3: *In vivo* confocal microscopy photographs showing cellular features in the corneal epithelial basement layer of different stages of *Streptococcal* keratitis. (A): Stage I; (B): Stage II; (C): Stage III.

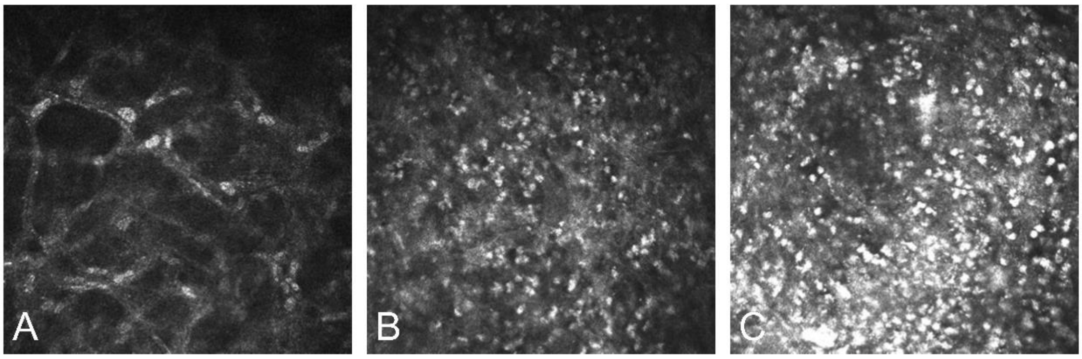

Figure S4: ***In vivo*** **confocal microscopy photographs showing cellular features in the corneal anterior stroma layer of different stages of** ***Streptococcal*** **keratitis.** (A): Stage I; (B): Stage II; (C): Stage III.

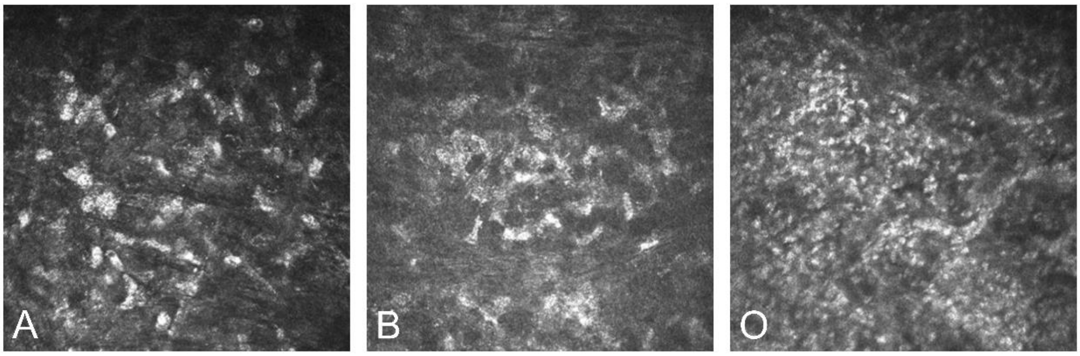

Figure S5: ***In vivo*** **confocal microscopy photographs showing cellular features in the corneal posterior stroma layer of different stages of** ***Streptococcal*** **keratitis.** (A): Stage I; (B): Stage II; (C): Stage III.

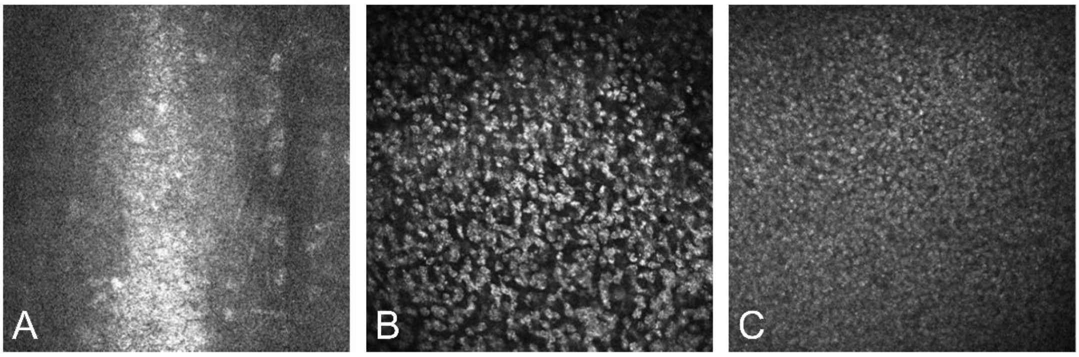

Figure S6: ***In vivo*** **confocal microscopy photographs showing cellular features in the corneal endothelial layer and post-corneal region of different stages of** ***Streptococcal*** **keratitis.** (A): Stage I; (B): Stage II; (C): Stage III.

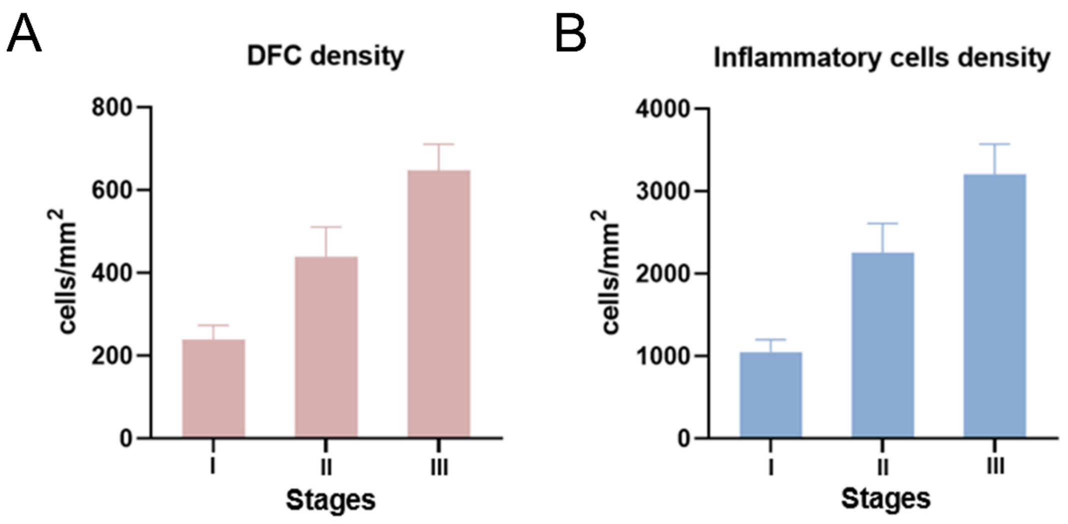

Figure S7: Inflammatory cell density and dendritiform cell density changes during the progression of *Streptococcal* keratitis. (A) Inflammatory cell density; (B) dendritiform cell density.

Table S1. Clinical and laboratory standards institute (CLSI) breakpoint standards for antimicrobial susceptibility testing of antibiotics.

Antimicrobial Agents	Disk content	Interpretive Categories and Zone Diameter Breakpoints		
		S	I	R
Vancomycin	30μg	≥17	-	-
Amikacin	30μg	≥17	15-16	≤14
Gentamicin	10μg	≥15	13-14	≤12
Ciprofloxacin	5μg	≥17	14-16	≤13
Ofloxacin	5μg	≥16	13-15	≤12
Benzalkonium chloride	1μg	≥22	-	-
Levofloxacin	5μg	≥17	14-16	≤13
Tobramycin	10μg	≥15	13-14	≤12
Ceftazidime	30μg	≥18	15-17	≤14
Moxifloxacin	5μg	≥18	15-17	≤14
Rifampin	5μg	≥20	17-19	≤16

Note: S: Susceptible, I: Intermediate, and R: Resistant, represent the levels of antimicrobial susceptibility.

Table S2. Antimicrobial Susceptibility Results of isolated Streptococcal Strains.

Streptococcus Species	Antimicrobial Resistance	Vancomycin	Ceftazidime	Ofloxacin	Levofloxacin	Rifampin	Moxifloxacin	Ciprofloxacin	Benzalkonium chloride	Amikacin	Gentamycin	Tobramycin
S.pneumoniae	S	86(98.9)	44(50.6)	75(86.2)	82(94.3)	83(95.4)	75(86.2)	3(3.4)	40(95.2)	5(5.7)	19(21.8)	11(12.6)
n(%)	I	0(0.0)	6(6.9)	7(8.0)	3(3.4)	3(3.4)	0(0.0)	5(5.7)	0(0.0)	3(3.4)	6(6.9)	3(3.4)
	R	1(1.1)	37(42.5)	5(5.8)	2(2.3)	1(1.1)	12(13.8)	79(90.8)	2(4.8)	79(90.8)	62(71.3)	73(83.9)
S.oralis	S	66(100.0)	13(19.7)	43(65.2)	50(75.8)	64(97.0)	54(81.8)	44(66.7)	24(82.8)	4(6.1)	21(31.8)	5(8.3)
n(%)	I	0(0.0)	8(12.1)	4(6.1)	2(3.0)	2(3.0)	4(6.1)	3(4.5)	0(0.0)	6(9.1)	5(7.6)	1(1.5)
	R	0(0.0)	45(68.2)	19(28.8)	14(21.2)	0(0.0)	8(12.1)	19(28.8)	5(17.2)	56(84.8)	40(60.6)	60(90.9)
S.mitis	S	71(97.3)	26(35.6)	51(69.9)	55(75.3)	69(94.5)	59(80.8)	50(68.5)	34(77.3)	12(16.4)	28(38.4)	9(12.3)
n(%)	I	0(0.0)	12(16.4)	6(8.2)	4(5.5)	2(2.7)	5(6.8)	6(8.2)	0(0.0)	10(13.7)	13(17.8)	6(8.2)
	R	2(2.7)	35(47.9)	16(21.9)	14(19.2)	2(2.7)	9(12.3)	17(23.3)	10(22.7)	51(69.8)	32(43.8)	58(79.5)
S.sanguis	S	21(100)	14(66.7)	17(81.0)	18(85.7)	18(85.7)	19(90.5)	16(76.2)	17(81.0)	5(23.8)	12(57.1)	10(47.6)
n(%)	I	0(0.0)	5(23.8)	0(0.0)	1(4.8)	1(4.8)	1(4.8)	2(9.5)	0(0.0)	5(23.8)	3(14.3)	0(0.0)
	R	0(0.0)	2(9.5)	4(19.0)	2(9.5)	2(9.5)	1(4.8)	3(14.3)	4(19.0)	11(52.4)	6(28.6)	11(52.4)
S.constellatus	S	5(100.0)	4(80.0)	5(100.0)	5(100.0)	5(100.0)	4(80.0)	4(80.0)	4(80.0)	4(80.0)	4(80.0)	4(80.0)
n(%)	I	0(0.0)	0(0.0)	0(0.0)	0(0.0)	0(0.0)	0(0.0)	1(20.0)	0(0.0)	0(0.0)	0(0.0)	0(0.0)
	R	0(0.0)	1(20.0)	0(0.0)	0(0.0)	0(0.0)	1(20.0)	0(0.0)	1(20.0)	1(20.0)	1(20.0)	1(20.0)
S.salivarius	S	4(80.0)	5(100.0)	3(60.0)	5(100.0)	4(80.0)	4(80.0)	3(60.0)	2(40.0)	0(0.0)	2(40.0)	0(0.0)
n(%)	I	0(0.0)	0(0.0)	2(40.0)	0(0.0)	1(20.0)	1(20.0)	2(40.0)	0(0.0)	0(0.0)	0(0.0)	0(0.0)
	R	1(20.0)	0(0.0)	0(0.0)	0(0.0)	0(0.0)	0(0.0)	0(0.0)	3(60.0)	5(100.0)	3(60.0)	5(100.0)
S.agalactiae	S	1(25.0)	3(75.0)	0(0.0)	0(0.0)	3(75.0)	1(25.0)	0(0.0)	1(25.0)	1(25.0)	0(0.0)	2(50.0)
n(%)	I	0(0.0)	0(0.0)	1(25.0)	1(25.0)	1(25.0)	0(0.0)	1(25.0)	0(0.0)	1(25.0)	1(25.0)	0(0.0)
	R	3(75.0)	1(25.0)	3(75.0)	3(75.0)	0(0.0)	3(75.0)	3(75.0)	3(75.0)	2(50.0)	3(75.0)	2(50.0)
S.dysgalactie	S	1(25.0)	3(75.0)	1(25.0)	3(75.0)	3(75.0)	3(75.0)	2(50.0)	2(50.0)	0(0.0)	1(25.0)	1(25.0)

n(%)	I	0(0.0)	0(0.0)	2(50.0)	1(25.0)	1(25.0)	1(25.0)	2(50.0)	0(0.0)	1(25.0)	0(0.0)	1(25.0)
	R	3(75.0)	1(25.0)	1(25.0)	0(0.0)	0(0.0)	0(0.0)	0(0.0)	2(50.0)	3(75.0)	3(75.0)	2(50.0)
S.goronii	S	1(25.0)	3(75.0)	1(25.0)	3(75.0)	3(75.0)	3(75.0)	2(50.0)	2(50.0)	0(0.0)	1(25.0)	1(25.0)
n(%)	I	0(0.0)	0(0.0)	2(50.0)	1(25.0)	1(25.0)	1(25.0)	2(50.0)	0(0.0)	1(25.0)	0(0.0)	1(25.0)
	R	3(75.0)	1(25.0)	1(25.0)	0(0.0)	0(0.0)	0(0.0)	0(0.0)	2(50.0)	3(75.0)	3(75.0)	2(50.0)
S.anginosus	S	4(100.0)	4(100.0)	4(100.0)	3(75.0)	3(75.0)	4(100.0)	3(75.0)	2(50.0)	0(0.0)	2(50.0)	1(25.0)
n(%)	I	0(0.0)	0(0.0)	0(0.0)	1(25.0)	0(0.0)	0(0.0)	1(25.0)	0(0.0)	1(25.0)	0(0.0)	0(0.0)
	R	0(0.0)	0(0.0)	0(0.0)	0(0.0)	1(25.0)	0(0.0)	0(0.0)	2(50.0)	3(75.0)	2(50.0)	3(75.0)
S.pyogenes	S	4(100.0)	3(75.0)	3(75.0)	3(75.0)	4(100.0)	3(75.0)	3(75.0)	3(75.0)	1(25.0)	2(50.0)	1(25.0)
n(%)	I	0(0.0)	1(25.0)	0(0.0)	0(0.0)	0(0.0)	1(25.0)	1(25.0)	0(0.0)	0(0.0)	0(0.0)	0(0.0)
	R	0(0.0)	0(0.0)	1(25.0)	1(25.0)	0(0.0)	0(0.0)	0(0.0)	1(25.0)	3(75.0)	2(50.0)	3(75.0)
Others	S	3(100.0)	3(100.0)	3(100.0)	3(100.0)	3(100.0)	3(100.0)	3(100.0)	2(66.7)	1(33.3)	2(66.7)	2(66.7)
n(%)	I	0(0.0)	0(0.0)	0(0.0)	0(0.0)	0(0.0)	0(0.0)	0(0.0)	0(0.0)	0(0.0)	0(0.0)	0(0.0)
	R	0(0.0)	0(0.0)	0(0.0)	0(0.0)	0(0.0)	0(0.0)	0(0.0)	1(33.3)	2(66.7)	1(33.3)	1(33.3)

Note: S: Susceptible, I: Intermediate, and R: Resistant, represent the levels of antimicrobial susceptibility.

Table S3. Microorganisms involved in mixed Infections in patients with *Streptococcal* keratitis.

**Pathogens**	**Number**
**Bacteria**	16
Gram positve	8
Staphylococcus	5
Corynebacterium	3
Gram negative	8
Pseudomonas	3
Klebsiella	2
Acinetobacter	2
Haemophilus	1
**Fungi**	11
Fusarium	4
Aspergillus	3
Alternaria	2
Candida	1
Penicillium	1
**Parasites**	2
Acanthamoeba	2
**Total**	29
